# Exploring optimum cut-off scores to screen for probable posttraumatic stress disorder within a sample of UK treatment-seeking veterans

**DOI:** 10.1080/20008198.2017.1398001

**Published:** 2017-11-13

**Authors:** Dominic Murphy, Jana Ross, Rachel Ashwick, Cherie Armour, Walter Busuttil

**Affiliations:** ^a^ Research Department, Combat Stress, Leatherhead, UK; ^b^ King’s Centre for Military Health Research, Department of Psychological Medicine, King’s College London, London, UK; ^c^ Faculty of Life & Health Sciences, Psychology Research Institute, Ulster University, Coleraine, Northern Ireland, UK

**Keywords:** Veterans, military, PTSD, CAPS, PCL-5, IES-R, psychometrics, veteranos, militar, TEPT, CAPS, PCL-5, IES-R, psicometría, 老兵，军队，PTSD，CAPS，PCL-5，IES-R，心理测量, • The psychometric properties of two measures of PTSD (PCL-5 & IES-R) were validated against a gold standard measure of PTSD (CAPS-5).• Good overall accuracy was observed for identifying PTSD positive cases for these measures.• Optimal cut-offs to indicate probable PTSD were observed to be higher than previously recommended.• Some discrepancy was found between identifying cases using measures based on the DSM-IV (IES-R) and DSM-5 (PCL-5).

## Abstract

**Background**: Previous research exploring the psychometric properties of the scores of measures of posttraumatic stress disorder (PTSD) suggests there is variation in their functioning depending on the target population. To date, there has been little study of these properties within UK veteran populations.

**Objective**: This study aimed to determine optimally efficient cut-off values for the Impact of Event Scale-Revised (IES-R) and the PTSD Checklist for DSM-5 (PCL-5) that can be used to assess for differential diagnosis of presumptive PTSD.

**Methods**: Data from a sample of 242 UK veterans assessed for mental health difficulties were analysed. The criterion-related validity of the PCL-5 and IES-R were evaluated against the Clinician-Administered PTSD Scale for DSM-5 (CAPS-5). Kappa statistics were used to assess the level of agreement between the DSM-IV and DSM-5 classification systems.

**Results**: The optimal cut-off scores observed within this sample were 34 or above on the PCL-5 and 46 or above on the IES-R. The PCL-5 cut-off is similar to the previously reported values, but the IES-R cut-off identified in this study is higher than has previously been recommended. Overall, a moderate level of agreement was found between participants screened positive using the DSM-IV and DSM-5 classification systems of PTSD.

**Conclusions**: Our findings suggest that the PCL-5 and IES-R can be used as brief measures within veteran populations presenting at secondary care to assess for PTSD. The use of a higher cut-off for the IES-R may be helpful for differentiating between veterans who present with PTSD and those who may have some sy`mptoms of PTSD but are sub-threshold for meeting a diagnosis. Further, the use of more accurate optimal cut-offs may aid clinicians to better monitor changes in PTSD symptoms during and after treatment.

## Abstract (es)

**Planteamiento**: La investigación previa que exploró las propiedades psicométricas de las puntuaciones de las medidas del trastorno de estrés postraumático (TEPT) sugiere que existe una variación en su funcionamiento según la población objetivo. Hasta la fecha, ha habido pocos estudios sobre estas propiedades dentro de las poblaciones de veteranos del Reino Unido.

**Objetivo**: Este estudio tuvo como objetivo determinar valores de corte óptimamente eficientes para la escala revisada del impacto de los eventos (IES-R, siglas en inglés de Impact of Event Scale-Revised) y la lista de TEPT para el DSM-5 (PCL-5, siglas en inglés de PTSD Checklist for DSM-5) que pueden utilizarse para evaluar el diagnóstico diferencial de TEPT presuntivo.

**Métodos**: Se analizaron los datos de una muestra de 242 veteranos del Reino Unido evaluados por dificultades de salud mental. La validez relacionada con los criterios del PCL-5 y la IES-R se evaluó frente a la Escala de TEPT administrada por el clínico para el DSM-5 (CAPS-5, siglas en inglés de Clinician-Administered PTSD Scale for DSM-5). Se usaron estadísticas Kappa para evaluar el nivel de acuerdo entre los sistemas de clasificación del DSM-IV y DSM-5.

**Resultados**: Las puntuaciones de corte óptimos observadas dentro de esta muestra fueron de 34 o más en la PCL-5 y de 46 o más en la IES-R. El límite de la PCL-5 es similar a los valores informados anteriormente, pero el punto de corte IES-R identificado en este estudio es más alto de lo que se había recomendado anteriormente. En general, se encontró un nivel moderado de acuerdo entre los participantes seleccionados con los sistemas de clasificación DSM-IV y DSM-5 para el TEPT.

**Conclusiones**: Nuestros hallazgos sugieren que la PCL-5 y la IES-R pueden usarse como medidas breves en poblaciones de veteranos que se presentan en el sistema sanitario secundario para evaluar el TEPT. El uso de un punto de corte más alto para la IES-R puede ser útil para diferenciar entre los veteranos que presentan TEPT y aquellos que pueden presentar algunos síntomas de TEPT, pero que están por debajo del umbral y no cumplen con el diagnóstico. Además, el uso de puntos de corte óptimos más precisos puede ayudar a los clínios a controlar mejor los cambios en los síntomas de TEPT durante y después del tratamiento.

## Abstract (zh)

**背景**：探索创伤后应激障碍（PTSD）测量分数的心理测量特性的前人研究建议，根据不同目标群体测量分数的表现具有变异性。目前为止，很少有研究在英国老兵群体里考察这些特性。

**目标**：本研究目的是要决定事件影响量表修订版（IES-R）和DSM-5 PTSD检核表（PCL-5）的划界分，用来评估不同PTSD诊断。

**方法**：使用242名英国老兵评估精神健康的数据，将PCL-5和IES-R与CAPS-5对比得出诊断标准相关效度。使用Kappa系数来评估DSM-IV和DSM-5分类系统的一致性。

**结果**：本样本中PCL-5最好的划界分是34或以上，IES-R是46或以上。PCL-5划界分和之前报告值相近，但是IES-R则高于以往推荐的数值。总体上，使用DSM-IV和DSM-5的PTSD分类系统表现出中等一致性。

**结论**：我们的发现建议PCL-5和IES-R可以用在二级护理的老兵群体中作为评估PTSD的简要工具。使用更高的IES-R划界分可以有助于区分有PTSD和可能具有一些症状但还在达不到PTSD诊断阈值的老兵。进一步，使用更准备的最佳划界分可能帮助临床医生更好监控PTSD症状在治疗中和治疗后的改变。

## 1. Introduction

A range of self-report questionnaires have been developed to assess for the presence of posttraumatic stress disorder (PTSD) and for the severity of PTSD symptoms. These measures often utilize cut-off scores to indicate presumptive PTSD. An examination of the literature suggests that there is much variation in the recommended cut-offs. For example, Keen et al. (Keen, Kutter, Niles, & Krinslet, 2008) observed that cut-offs for the PTSD Checklist (PCL-C) (one of the most frequently used self-report questionnaires) for probable DSM-IV PTSD ranged from 28 to 50 (Dobie et al., 2002; Forbes, Creamer, & Biddle, 2001; Lang, Laffaye, Satz, Dresselhaus, & Stein, 2003; Yeager, Magruder, Knapp, Nicholas, & Frueh, 2007). Cut-off values have been reported as high as 60 (Keen et al., 2008). Taken together, the results suggest a high variability in the proposed cut-off values and point to the possibility that different cut-offs may be appropriate in different populations. In general there appears to be a trend for studies sampling from veteran populations to report higher optimal cut-offs than those conducted using non-military samples (Keen et al., 2008; McDonald & Calhoun, 2010).

A further observation is that the majority of the studies that have explored the psychometric properties of PTSD measures within veteran populations have done so by recruiting from either community populations or primary care mental health settings (Creamer, Bell, & Failla, 2003; Hoge, Riviere, Wilk, Herrell, & Weather, 2014; Keen et al., 2008). As such, this may limit the generalizability of these recommendations when applying to treatment-seeking veterans in secondary care settings. Indeed, it has been suggested that the purpose of the test (screening versus making a diagnosis), the population under investigation (community populations versus those recruited in a clinical setting, heterogeneity of population and prevalence of PTSD within target population) and how the identification of optimal cut-offs have been defined statistically need to be taken into consideration when deciding on optimal cut-offs (Foa et al., 2016; Keen et al., 2008).

The Clinician-Administered PTSD scale (CAPS) is the most widely used and accepted criterion measure of PTSD (Weathers, Keane, & Davidson, 2001; Weathers, Ruscio, & Keane, 1999). However, the CAPS must be administered by a trained professional and can be time consuming to complete. As such, the use of concise self-report questionnaires can be helpful to facilitate data collection and monitor outcomes. As discussed above, there seems little consensus on the optimal cut-offs for clinical populations of veterans which suggests the need to validate measures within the target population rather than generalizing from other population groups. The aforementioned limitations may restrict the applicability of using existing cut-offs to suggest presumptive PTSD for veterans within secondary care settings. For example, veterans within these settings tend to present with a high burden of PTSD symptomatology, along with a range of other co-morbid mental health difficulties, functional impairment and evidence of childhood adversity (Murphy et al., 2015). Further, there is a paucity of research that explores appropriate cut-offs for probable PTSD on a range of screening psychometric measures within UK treatment-seeking veteran populations, with the majority of research coming from the US. The identification of these cut-offs could be helpful for both researchers and clinicians working within this field.

## 2. Objective

The aim of this study was to identify the optimally efficient cut-off scores on two self-report questionnaires for PTSD against the Clinician-Administered PTSD Scale for DSM-5 (CAPS-5) within a sample of treatment-seeking UK veterans recruited from a secondary care setting. The study aimed to identify cut-off values for making a differential diagnosis of PTSD. This was done by collecting data during clinical assessments that were conducted at a national charity in the UK which supports veterans with mental health difficulties.

## 3. Method

### 3.1. Procedure

Data was collected from a national charity in the UK called Combat Stress (CS) that offers clinical services to veterans with mental health difficulties. As part of the referral process to CS, individuals were triaged by a nurse and then those identified as experiencing mental health difficulties, using a standardized clinical interview, were offered a formal assessment to screen for presence of PTSD and other mental health difficulties. These assessments were conducted by certified Clinical Psychologists and CBT therapists. Therapists had all been trained by the same psychiatrist on how to administer the CAPS-5 and further supervision was provided aimed at improving fidelity. During this assessment a standard set of psychometric measures were administered. The PTSD Checklist for DSM-5 (PCL-5) and Impact of Event Scale-Revised (IES-R) were counter-balanced with approximately half the packs of measures having the PCL-5 at the start and the IES-R at the end and vice versa. Data was collected over a six-month period from February 2016 to July 2016 from 223 participants. In addition, we included data from 23 individuals who had already been assessed in the three months prior to February 2016, had completed the standardized batch of psychometric measures and subsequently been screened negative for PTSD on the PCL-5. The rationale for this was to ensure there was sufficient variation in PTSD caseness and severities to be able to conduct statistical analysis (Hajian-Tilaki, 2013). Whilst this was not ideal, data was collected in an identical manner for these 23 participants as they had been recruited from consecutive assessments in which individuals had been screened as not meeting criteria for PTSD.

### 3.2. Participants

In total, 246 participants were recruited into the study. Inclusion criteria were being a veteran (in the UK this equates to having completed one full day of employment in the Armed Forces; Dandeker, Wessely, Iversen, & Ross, 2006) and having been assessed by CS’s triage nurses as having a mental health difficulty. Exclusion criteria included being actively psychotic at the time of the assessment interview or being in a state of intoxication during the assessment. We made effort to ensure participant recruitment was consistent with second version of the Quality Assessment of Diagnostic Accuracy Studies (QUADAS-2) guidance (Whiting et al., 2011).

### 3.3. Measures

Participants were asked to self-complete two psychometric measures (IES-R and PCL-5). The assessor then administered the CAPS-5. Whilst the IES-R does not directly overlap with the DSM-IV PTSD, it assesses for three clusters of symptoms that map onto the three main DSM-IV symptom criterions B, C and D for PTSD. The other two measures assessed the DSM-5 PTSD symptoms. Socio-demographic characteristics were also collected. These included sex, age, educational achievement and current employment status.

#### 3.3.1. Clinician-Administered PTSD Scale for DSM-5

The CAPS-5 is a structured clinical interview that includes 30 items (Weathers et al., 2013a). Twenty of these items assess for the presence of the 20 PTSD symptoms as outlined in the DSM-5. In addition, 10 items explore the onset of symptoms, duration of symptoms, subjective distress, functional impairment and information on dissociative symptoms. The interviewer evaluates the intensity and frequency of PTSD symptoms and then combines these according to explicit scoring rules to identify the appropriate severity rating. For each item there are five rating scale options for rating symptom severity score 0–4 (absent/mild/moderate/severe/extreme). In addition, there are four choices for rating symptom frequency (minimal/clearly present/pronounced/extreme). For a symptom to meet threshold for being present it needed to receive a score of two or above for severity and two or above for frequency. The CAPS-5 can be used to diagnose PTSD if respondents endorse symptoms from each of the criteria as set out within the DSM-5 (Weathers et al., 2013a). To do so, participants had to meet criterion A (exposure to an actual or threatened death, serious injury or sexual violence), endorse one symptom from criterion B (intrusion symptoms), one symptom from criterion C (avoidance symptoms), two symptoms from criterion D (cognition or mood symptoms), two symptoms from criterion E (arousal and reactivity symptoms), meet criterion F (duration of disturbance for longer than one month) and report one symptoms from criterion G (distress or impairment).

#### 3.3.2. PTSD Checklist for DSM-5

The PCL-5 is a 20-item self-report measure that assesses for the presence of the 20 DSM-5 symptoms of PTSD. It has been suggested for use as a screening tool and for making a diagnosis of probable PTSD. Each item asks about how much a particular symptom has bothered the individual over the previous month and then gives five options (ranging from ‘not at all’ to ‘extremely’) scored 0–4. Total scores can range from 0 to 80. Initial work has suggested a cut-off of either 33 or 38 for veterans being screened for symptoms of PTSD (Bovin et al., 2016; Hoge et al., 2014; Weathers et al., 2013b; Wortmann et al., 2016).

#### 3.3.3. Impact of Event Scale-Revised

The IES-R is a 22-item self-report measure assessing the presence of PTSD symptom clusters that map onto the DSM-IV criteria (Creamer et al., 2003). Individuals are asked to rate how distressing different symptoms of PTSD have been for them over the past seven days. Five response choices are available (ranging from ‘not at all’ to ‘extremely’) and are scored 0–4. Total scores can range from 0 to 88. Higher total scores are suggestive of more severe presentations. The IES-R is widely used in primary care psychology services in the UK. A cut-off for probable PTSD according to DSM-IV for veterans has been suggested to be a score of 33 or above (Creamer et al., 2003).

### 3.4. Analysis

Participants who had not completed one or more measures (CAPS-5, PCL-5 or IES-R) were excluded from the analysis (*n* = 4). For the remaining participants, no missing data was present for individual items from these measures. Following Kraemer’s (1987) guidelines, signal detection analyses (Quality Receiver Operating Characteristics; QROC) were conducted to establish optimally efficient cut-off scores on PCL-5 and IES-R relative to the CAPS-5 diagnosis (Kraemer, 1987). Kraemer (1987) recommended the use of recalibrated statistics (sensitivity, specificity, efficiency) when deciding on the optimal cut-off scores. Such recalibrated statistics are chance-corrected, which means that the possibility of obtaining high values with chance classifications is reduced. Typically, three measures of recalibrated Cohen’s kappa statistics are used depending on whether the primary focus is on: (1) screening or false negatives (ĸ(1) or recalibrated sensitivity); (2) definitive tests or false positives (ĸ(0) or recalibrated specificity); or (3) differential diagnosis or equal concern with false positives and false negatives (ĸ(0.5) or recalibrated efficiency). ĸ(0) represents the quality of specificity, ĸ(1) represents the quality of sensitivity and ĸ(0.5) represents the quality of efficiency. In the current study, the focus was on differential diagnosis (i.e. quality of efficiency or ĸ(0.5)). Values of ĸ(0.5) can range between 0 and 1, with 0 indicating no agreement (other than that which would be obtained by chance) and 1 indicating total agreement. The recalibrated kappa statistics are concerned with the quality of diagnostic tests. Measures of the performance of diagnostic tests, such as uncalibrated sensitivity, specificity, efficiency, positive and negative predictive values and Youden’s index, were also calculated.

The area under the ROC curve (AUC) and its associated 95% confidence intervals were calculated for each test using the DeLong method. The AUC value can vary between 0.5 and 1 and represents the overall accuracy of the diagnostic test in predicting diagnostic caseness (Faraggi & Reiser, 2002). Values of 1 indicate perfect accuracy (i.e. 100% sensitive and 100% specific), whereas values of 0.5 indicate no discriminatory power (i.e. 50% sensitive and 50% specific).

In the final stage of analysis, kappa statistics were run to explore the level of agreement between meeting case criteria on the PCL-5 and the IES-R . This was run three times; twice using the previously recommended cut-offs and then once again using optimal cut-offs identified within the current study. The PCL-5 and IES-R were chosen for this analysis to explore the level of agreement between PTSD cases as identified by the DSM-IV and DSM-5 when using self-completed measures. All basic analyses were conducted in SPSS 23. The QROC analyses were performed using Excel spreadsheets. Receiver Operating Characteristic (ROC) curves were fitted using the pROC package (Robin et al., 2011) version 1.9.1 in the R environment.

## 4. Results

Of the 246 participants, four did not complete the IES-R and were excluded from the analysis, leaving an effective sample of 242 participants who had no missing data. Data presented in Table 1 using the QUADAS-2 domains suggest that the majority of the criteria for high levels of generalizability were met. Demographic information of the effective sample is presented in Table 2. A total of 77.7% (*n* = 188) of the 242 participants met the DSM-5 diagnostic criteria for PTSD according to CAPS-5.

**Table 1.** QUADAS-2 domains, signalling questions and evaluation of the current study.

Domains	Signalling questions	Current study performance
Risk of bias domains		
Patient selection	Was a consecutive or random sample of patients enrolled?	Yes – consecutive sampling. Though 23 additional participants were added who have been retrospectively consecutively sampled from patients who screened negative to PTSD.
	Did the study avoid inappropriate exclusions?	Yes
	Was a case-control design avoided?	Yes
Index test	Were the index tests results interpreted without knowledge of the reference standard?	Yes
	If a threshold was used, was it pre-specified?	Yes
Reference standard	Is the reference standard likely to correctly classify the target disorder?	Yes – we used the gold standard CAPS-5
	Were the reference standard results interpreted without knowledge of the results of the index tests?	Yes
Flow and timing	Was there an appropriate interval between the index test and the reference standard?	Yes – data collected on the same day
	Did all participants receive the same reference standard?	Yes
	Were all patients included in the analysis?	No – four participants were excluded who had not completed one of the PTSD measures.
Applicability domains		
Patient selection	Are there concerns that the included patients and setting do not match the review question?	No – participants recruited from a clinical sample of veterans seeking services. The same setting for the review question
Index test	Are the concerns that the index tests, their conduct or their interpretation differ from the reviewer question?	No
Reference standard	Are there concerns that the target condition as defined by the reference standard does not match the question?	No

Note. A study that avoids all bias and has perfect generalizability answers ‘yes’ to all the bias domains signalling questions and ‘no’ to all the applicability domains signalling questions.

**Table 2.** Demographic information.

Variable	Effective sample (*N* = 242)
Gender, *n* (%)	
Male	237 (97.9)
Female	3 (1.2)
Age, *M* (*SD*)	44.0 (12.2)
Education, *n* (%)	
Left school	66 (27.3)
GCSE or equivalent^a^	89 (36.8)
A Level or equivalent^b^	42 (17.4)
Undergraduate degree or equivalent	21 (8.7)
Postgraduate degree or equivalent	4 (1.7)
Employment at time of assessment	
Full-time	92 (38.0)
Part-time	13 (5.4)
Not working	18 (7.4)
Not working due to ill health	71 (29.3)
Retired	20 (8.3)
Other	13 (5.4)

Note. Frequencies and percentages do not add up due to missing values.

^a^GCSE is education equivalent up to the age of 16.

^b^A Level is education equivalent up to the age of 18.

Following Kraemer’s (1987) guidelines for conducting QROC analyses, the optimally efficient cut-off score on PCL-5 relative to the CAPS-5 diagnosis was 34. It had the highest quality of efficiency index with a value of *ĸ*(0.5) = 0.52 (95% CI: 0.39–0.65), suggesting moderate agreement with CAPS-5. The associated uncalibrated sensitivity was 0.89 (95% CI: 0.85–0.94) and the uncalibrated specificity was 0.63 (95% CI: 0.50–0.76). The quality of sensitivity [*ĸ*(1)] and specificity [*ĸ*(0)] were both 0.52. The values corresponding to other tests of performance, including the positive predictive value, negative predictive value and efficiency, are presented in Supplemental data Table 1. Supplemental data Table 1 contains values for tests of performance and the associated values for tests of quality for a range of different PCL-5 scores. Youden’s index is also presented. Using the PCL-5 cut-off score of 34, 77.7% (*n *= 188) of the 242 participants would screen positive for PTSD (Positive Predictive Value (PPV) = 0.63; Negative Predictive Value (NPV) = 0.63).

The QROC analysis of the IES-R revealed an optimally efficient cut-off score of 46. The associated quality of efficiency index value was *ĸ*(0.5) = .51 (95% CI: 0.39–0.63), indicating moderate agreement with CAPS-5. The associated uncalibrated sensitivity was 0.83 (95% CI: 0.78–0.88) and the uncalibrated specificity was 0.74 (95% CI: 0.62–0.86). The quality of sensitivity [*ĸ*(1)] for this cut-off value was 0.43 and the quality of specificity [*ĸ*(0)] was 0.63. Supplemental data Table 2 contains the relevant values of different tests of performance and tests of quality for a range of IES-R scores. Using the cut-off score of 46, 70.2% (*n* = 170) of the 242 participants would screen positive for PTSD (PPV = 0.92; NPV = 0.56).

**Figure 1.**
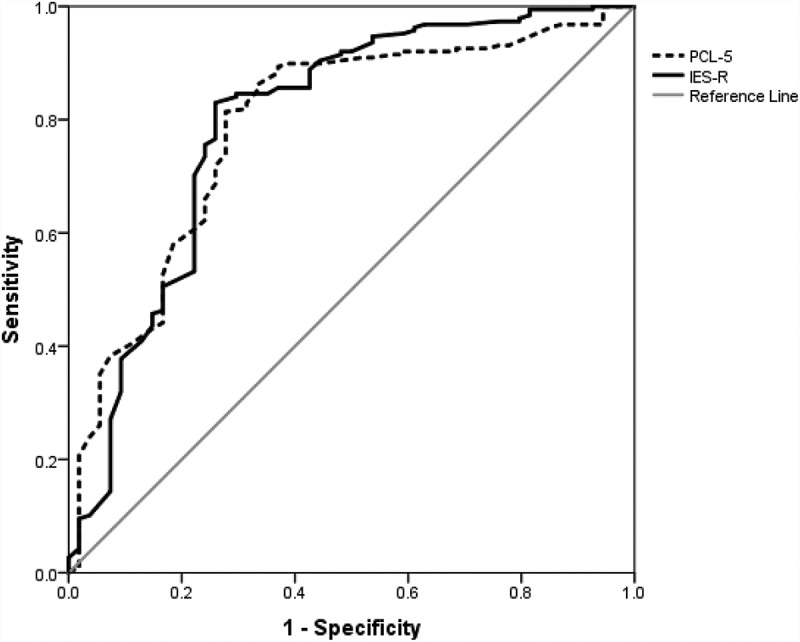
ROC curve for PCL-5 and IES-R in relation to the CAPS-5 PTSD diagnosis.

Table 3 presents the comparison of the optimal cut-off scores from the current study in relation to the cut-off scores recommended in the literature. The PCL-5 ROC curve analysis yielded an AUC value of 0.79 (95% CI: 0.72–0.86), indicating fair overall accuracy in predicting PTSD caseness. The IES-R ROC analysis revealed an AUC value of 0.80 (95% CI: 0.72–0.87), indicating fair to good accuracy in predicting PTSD caseness. The PCL-5 and IES-R AUCs were compared using the roc.test function from the pROC package and were found to be not significantly different from each other (*Z* = −0.21, *p* = .834). Both ROC curves are depicted in Figure 1.

**Table 3.** PTSD prevalence based on different measures and cut-off values.

Measure	Cut-off	*ĸ*(0)	*ĸ*(0.5)	*ĸ*(1)	PTSD prevalence
PCL-5	**34**	0.52	0.52	0.52	77.7%
	33^a,b^	0.51	0.52	0.53	78.5%
	38^c^	0.56	0.47	0.40	71.5%
IES-R	**46**	0.63	0.51	0.43	70.2%
	33^d^	0.40	0.46	0.54	82.6%

^a^Bovin et al. (2016). ^b^Wortmann et al. (2016). ^c^Weathers et al. (2013b). ^d^Creamer et al. (2003).

Cohen’s kappa statistics revealed a value of 0.50 (95%CI: 0.36–0.64, *p* < .001) between the previously recommended PCL-5 cut-off score of 33 and the IES-R cut-off score of 33. This represents observed agreement of 84.3% versus the expected agreement of 68.6% (Viera & Garrett, 2005). Using the previously recommended PCL-5 cut-off score of 38 and IES-R cut-off score of 33, Cohen’s kappa value was 0.53 (95% CI: 0.41–0.65, *p* < .001). Observed agreement was 83.1% and expected agreement was 64.0%. With the optimal cut-offs identified within this paper (i.e. 34 for PCL-5 and 46 for IES-R), the kappa value was 0.53 (95% CI: 0.41–0.65, *p* < .001), indicating moderate agreement. This represents observed agreement of 81.8% relative to the expected agreement of 61.2% (Viera & Garrett, 2005).

## 5. Discussion

The findings from this study showed that the psychometric measures of the PCL-5 and IES-R had fair to good accuracy in identifying PTSD cases as indicated by comparison against the CAPS-5 within a clinical sample of treatment-seeking UK veterans. There was a general trend towards identifying higher cut-off scores to indicate probable PTSD on these measures than had previously been recommended (Creamer et al., 2003; Foa, Riggs, Dancu, & Rothbaum, 1993; Keen et al., 2008).

The findings from the current study demonstrate the need to use higher cut-off on the IES-R than previously recommended when screening for PTSD within clinical samples of UK veterans seeking support for mental health difficulties. Our results suggest a score of 46 on the IES-R rather than the previous recommendation of 33 (Creamer et al., 2003). A very modest increase was noted for the PCL-5 compared the majority of previously recommended cut-offs, with a cut-off score of 34 compared to previous recommended score of 33; though it should be noted that 38 has also been suggested as a cut-off for the PCL-5 (Bovin et al., 2016; Weathers et al., 2013b; Wortmann et al., 2016). It is important to note that previous cut-offs been validated against the DSM-IV criteria rather than the DSM-5, as in the current study. This finding is supported by previous work that also indicated the need for higher cut-off values within veteran samples on a range of measures (Dobie et al., 2002; Foa et al., 2016; Forbes et al., 2001; Keen et al., 2008; Lang et al., 2003). This could have useful clinical implications when both assessing for the presence of PTSD, but also evaluating treatment outcomes. For example, data from a range of countries reporting on PTSD treatment outcomes in veterans suggest that, whilst significant reductions in the severity of symptoms are evident, many participants have scores on psychometric measures that still indicate meeting diagnostic criteria for PTSD (Creamer, Morris, Biddle, & Elliot, 1999; Currier, Holland, Drescher, & Elhai, 2014; Murphy et al., 2016; Richardson et al., 2014). It may be that the use of lower cut-offs, validated within different populations, could be masking positive treatment outcomes. Additionally, if used for screening, the use of lower established cut-offs may lead to the over diagnosis of PTSD in veterans.

A study conducted with Canadian military respondents found that a lower proportion of individuals screened positive for probable PTSD under the DSM-5 criteria compared to the DSM-IV criteria (71.2% vs 77.7%) using the PCL-M to define caseness against both the DSM-IV and DSM-5 (Roth, St Cyr, Levine, King, & Richardson, 2016). Similarly, within the current study, when we used the previously recommended cut-offs for the PCL-5 and IES-R we also observed a lower prevalence rate of PTSD under the DSM-5 criteria (78.5% vs 82.6%). However, the current study suggested optimal cut-offs for the PCL-5 and IES-R of 34 and 46 respectively. Using these cut-offs, the prevalence rate of probable PTSD as indicated by these measures was nearly identical (77.7% and 70.2%). This appears to suggest that changes in the criteria for PTSD between the DSM-IV and DSM-5 affected the overall prevalence rate more when using the IES-R than the PCL-5. Previous studies of US military have observed similar findings. Hoge et al. (2014) noted much variation in individuals who met criteria for PTSD under the DSM-IV or DSM-5 suggesting that, even though the overall proportions were similar, there were many cases of individuals meeting criteria under one classification system but not the other. Our findings also suggest there is variation in the individuals meeting case criteria between the two classification systems, with only moderate agreement between the PCL-5 and IES-R using previous recommended cut-offs. This did not change when using the optimal cut-offs identified within the current paper with a level of agreement of 81.8%. This indicated a moderate level of agreement and implies that many individuals are screening positive on one measure but not the other, and vice versa. Given the differences outlined above, and suggested difficulties with shift from the DSM-IV to the DSM-5 (Hoge et al., 2016; McFarlane, 2014), it would be prudent for more research to be conducted within UK military samples to aid clinicians, service providers and policy makers working within this field.

Due to a gap in the literature, the aim of the current study was to explore the utility of various brief PTSD screening measures within clinical populations of UK veterans. As such, the sample recruited should increase the ecological validity of our findings and improve generalizability. That said, there are a number of limitations that need to be considered when interpreting the results of the current study. Firstly, whilst the sample employed is appropriate to explore the utility of the PCL-5 and IES-R within treatment-seeking veterans, its lack of diversity may limit the generalizability of the findings to other populations, or possibly to epidemiological surveys of community veteran populations. Indeed, a previous review of the PTSD Checklist for DSM-IV suggested that cut-offs to indicate PTSD need to take into account both the population and function of the psychometric measure (Keen et al., 2008). Secondly, a further limitation may be that participants were recruited from a mental health service that specializes in treating PTSD. This may have introduced bias by influencing participants to recall potentially traumatic memories, or attribute current impairment to traumatic events, rather than other difficulties such as depression. Alternatively, there could have been a desire to receive a diagnosis as this could have been seen by participants as allowing them access to treatment. Previous qualitative research has indicated that a diagnosis of PTSD is seen as more acceptable to military personnel than a diagnosis of depression (Murphy, Hunt, Luzon, & Greenberg, 2014). As such, it is important to note that psychometric measures should not form the sole basis for a diagnosis. Thirdly, we were unable to explore test-retest reliability which may have increased confidence that the nature of the assessment had not influenced the completion of the psychometric measures. Fourthly, the CAPS-5 was administered by several different interviewers and it was not possible to assess inter-rater reliability for the CAPS-5. This was because the identities of the mental health professionals who conducted each CAPS-5 assessment were not recorded. As such, we were not able to quantify issues related to using multiple interviewers administering the CAPS-5. However, formal training for the CAPS-5, regular supervision and the use of only certified psychologists or CBT therapists to deliver it were in place to increase the administration fidelity.

Whilst the aim of this paper was to explore optimal cut-offs for the PCL-5 and IES-R for UK veterans, it is worth noting that there could be limitations to dichotomizing health outcomes. Harrell has written about a number of limitations that may result from dichotomizing continuous variables (Harrell, 2015). For example, whilst not exhaustive, these include the lack of power to detect changes in health presentations when using binary outcomes or the assumption that a cut-off is meaningful as PTSD symptoms may be better understood as being experienced on a continuum rather than being present or not present. Further, using a binary outcome means that severity of presentations cannot be assessed. For example, individuals who score one point above a cut-off for PTSD may present very differently clinically to individuals who score 30 points above the cut-off.

### 5.1. Conclusions

The data presented supports the use of these brief psychometric measures to assess for the presence of PTSD within clinical samples of UK veterans. In particular, the specificity and sensitivity scores for PCL-5 and IES-R suggest these are favourable measures that can be administered to veterans directly for them to complete themselves. Our data indicates the importance of using cut-offs that are appropriate to the target population. Appropriate cut-off scores for the PCL-5 and IES-R were found to be optimal at 34 and 46 respectively. For UK veterans seeking mental health support, the use of higher cut-off values appeared to improve the function of the tests.

## Supplementary Material

Supplementary material
