# Supplementary material for: Exploring optimum cut-off scores to screen for probable posttraumatic stress disorder within a sample of UK treatment-seeking veterans
Source: Eur J Psychotraumatol. 2017 Nov 13;8(1):1398001. doi: 10.1080/20008198.2017.1398001 (PMC5800736; doi:10.1080/20008198.2017.1398001)
Supplement: Supplementary material [file ZEPT_A_1398001_SM3472.docx]

*Supplementary Table 1. Diagnostic utility of a range of PCL-5 scores in relation to the CAPS-5 PTSD diagnosis (N = 242).*

| **Cut-off** | **Level** | **Sensitivity** | **95% CI (Sen)** | **Specificity** | **95% CI (Spe)** | **PPV** | **NPV** | **Efficiency** | **ĸ(0)** | **ĸ(0.5)** | **95% CI (ĸ(0.5))** | **ĸ(1)** | **Youden** |
| --- | --- | --- | --- | --- | --- | --- | --- | --- | --- | --- | --- | --- | --- |
| 20 ≤ | 0.87 | 0.92 | 0.88-0.96 | 0.31 | 0.19-0.44 | 0.82 | 0.53 | 0.79 | 0.21 | 0.27 | 0.13-0.42 | 0.40 | 0.24 |
| 21 ≤ | 0.86 | 0.92 | 0.88-0.96 | 0.33 | 0.21-0.46 | 0.83 | 0.55 | 0.79 | 0.23 | 0.29 | 0.15-0.44 | 0.41 | 0.25 |
| 22 ≤ | 0.85 | 0.92 | 0.88-0.96 | 0.39 | 0.26-0.52 | 0.84 | 0.58 | 0.80 | 0.28 | 0.35 | 0.21-0.49 | 0.46 | 0.31 |
| 23 ≤ | 0.85 | 0.92 | 0.88-0.96 | 0.41 | 0.28-0.54 | 0.84 | 0.59 | 0.81 | 0.30 | 0.37 | 0.23-0.51 | 0.48 | 0.33 |
| 24 ≤ | 0.84 | 0.91 | 0.88-0.95 | 0.41 | 0.28-0.54 | 0.84 | 0.58 | 0.80 | 0.30 | 0.36 | 0.22-0.50 | 0.46 | 0.32 |
| 25 ≤ | 0.84 | 0.91 | 0.88-0.95 | 0.41 | 0.28-0.54 | 0.84 | 0.58 | 0.80 | 0.30 | 0.36 | 0.22-0.50 | 0.46 | 0.32 |
| 26 ≤ | 0.84 | 0.91 | 0.88-0.95 | 0.43 | 0.29-0.56 | 0.85 | 0.59 | 0.81 | 0.32 | 0.38 | 0.24-0.52 | 0.47 | 0.34 |
| 27 ≤ | 0.83 | 0.91 | 0.87-0.95 | 0.46 | 0.33-0.60 | 0.86 | 0.60 | 0.81 | 0.35 | 0.40 | 0.26-0.55 | 0.48 | 0.37 |
| 28 ≤ | 0.83 | 0.91 | 0.87-0.95 | 0.46 | 0.33-0.60 | 0.86 | 0.60 | 0.81 | 0.35 | 0.40 | 0.26-0.55 | 0.48 | 0.37 |
| 29 ≤ | 0.82 | 0.91 | 0.87-0.95 | 0.48 | 0.35-0.61 | 0.86 | 0.60 | 0.81 | 0.37 | 0.42 | 0.28-0.56 | 0.49 | 0.39 |
| 30 ≤ | 0.81 | 0.90 | 0.86-0.95 | 0.52 | 0.39-0.65 | 0.87 | 0.61 | 0.82 | 0.41 | 0.45 | 0.31-0.58 | 0.50 | 0.42 |
| 31 ≤ | 0.81 | 0.90 | 0.86-0.95 | 0.52 | 0.39-0.65 | 0.87 | 0.61 | 0.82 | 0.41 | 0.45 | 0.31-0.58 | 0.50 | 0.42 |
| 32 ≤ | 0.80 | 0.90 | 0.86-0.94 | 0.56 | 0.42-0.69 | 0.88 | 0.61 | 0.82 | 0.44 | 0.47 | 0.33-0.61 | 0.50 | 0.45 |
| 33 ≤ | 0.79 | 0.90 | 0.86-0.94 | 0.61 | 0.48-0.74 | 0.89 | 0.63 | 0.83 | 0.50 | 0.52 | 0.39-0.65 | 0.53 | 0.51 |
| **34 ≤** | **0.78** | **0.89** | **0.85-0.94** | **0.63** | **0.50-0.76** | **0.89** | **0.63** | **0.83** | **0.52** | **0.52** | **0.39-0.65** | **0.52** | **0.52** |
| 35 ≤ | 0.77 | 0.88 | 0.84-0.93 | 0.63 | 0.50-0.76 | 0.89 | 0.61 | 0.83 | 0.52 | 0.51 | 0.38-0.64 | 0.49 | 0.51 |
| 36 ≤ | 0.74 | 0.86 | 0.81-0.91 | 0.67 | 0.54-0.79 | 0.90 | 0.58 | 0.82 | 0.55 | 0.50 | 0.37-0.63 | 0.46 | 0.53 |
| 37 ≤ | 0.74 | 0.85 | 0.80-0.90 | 0.67 | 0.54-0.79 | 0.90 | 0.56 | 0.81 | 0.55 | 0.49 | 0.36-0.61 | 0.44 | 0.52 |
| 38 ≤ | 0.71 | 0.83 | 0.77-0.89 | 0.69 | 0.56-0.81 | 0.90 | 0.54 | 0.80 | 0.56 | 0.47 | 0.34-0.59 | 0.40 | 0.51 |
| 39 ≤ | 0.71 | 0.82 | 0.76-0.88 | 0.69 | 0.56-0.81 | 0.90 | 0.52 | 0.79 | 0.55 | 0.45 | 0.33-0.58 | 0.38 | 0.50 |
| 40 ≤ | 0.69 | 0.81 | 0.75-0.87 | 0.72 | 0.60-0.84 | 0.91 | 0.53 | 0.79 | 0.60 | 0.47 | 0.35-0.60 | 0.39 | 0.54 |
| 41 ≤ | 0.69 | 0.81 | 0.75-0.87 | 0.72 | 0.60-0.84 | 0.91 | 0.52 | 0.79 | 0.60 | 0.47 | 0.34-0.59 | 0.38 | 0.53 |
| 42 ≤ | 0.69 | 0.80 | 0.74-0.86 | 0.72 | 0.60-0.84 | 0.91 | 0.51 | 0.79 | 0.60 | 0.46 | 0.34-0.58 | 0.37 | 0.53 |
| 43 ≤ | 0.67 | 0.79 | 0.72-0.85 | 0.72 | 0.60-0.84 | 0.91 | 0.49 | 0.77 | 0.59 | 0.44 | 0.32-0.56 | 0.35 | 0.51 |
| 44 ≤ | 0.64 | 0.75 | 0.68-0.82 | 0.72 | 0.60-0.84 | 0.90 | 0.45 | 0.74 | 0.57 | 0.39 | 0.27-0.51 | 0.30 | 0.47 |
| 45 ≤ | 0.64 | 0.74 | 0.67-0.81 | 0.72 | 0.60-0.84 | 0.90 | 0.44 | 0.74 | 0.56 | 0.38 | 0.26-0.50 | 0.28 | 0.46 |
| 46 ≤ | 0.62 | 0.72 | 0.65-0.79 | 0.74 | 0.62-0.86 | 0.91 | 0.43 | 0.72 | 0.58 | 0.36 | 0.25-0.48 | 0.27 | 0.46 |
| 47 ≤ | 0.59 | 0.69 | 0.61-0.76 | 0.74 | 0.62-0.86 | 0.90 | 0.40 | 0.70 | 0.56 | 0.33 | 0.22-0.44 | 0.23 | 0.43 |
| 48 ≤ | 0.57 | 0.66 | 0.58-0.74 | 0.76 | 0.65-0.87 | 0.91 | 0.39 | 0.68 | 0.57 | 0.31 | 0.20-0.43 | 0.22 | 0.42 |
| 49 ≤ | 0.56 | 0.65 | 0.57-0.73 | 0.76 | 0.65-0.87 | 0.90 | 0.38 | 0.67 | 0.57 | 0.30 | 0.19-0.41 | 0.21 | 0.41 |
| 50 ≤ | 0.55 | 0.63 | 0.55-0.72 | 0.76 | 0.65-0.87 | 0.90 | 0.37 | 0.66 | 0.56 | 0.29 | 0.18-0.39 | 0.19 | 0.39 |

*Note*. Level = Level of test (% of participants meeting the cut-off); 95% CI (Sen) = 95% confidence interval for sensitivity; 95% CI (Spe) = 95% confidence interval for specificity; PPV = Positive predictive value; NPV = Negative predictive value; ĸ(0) = Quality of specificity; ĸ(0.5) = Quality of efficiency; 95% CI (ĸ(0.5)) = 95% confidence interval for the quality of efficiency; ĸ(1) = Quality of sensitivity; Youden = Youden’s index.

*Supplementary Table 2. Diagnostic utility of a range of IES-R scores in relation to the CAPS-5 PTSD diagnosis (N = 242).*

| **Cut-off** | **Level** | **Sensitivity** | **95% CI (Sen)** | **Specificity** | **95% CI (Spe)** | **PPV** | **NPV** | **Efficiency** | **ĸ(0)** | **ĸ(0.5)** | **95% CI (ĸ(0.5))** | **ĸ(1)** | **Youden** |
| --- | --- | --- | --- | --- | --- | --- | --- | --- | --- | --- | --- | --- | --- |
| 25 ≤ | 0.90 | 0.97 | 0.94-0.99 | 0.35 | 0.22-0.48 | 0.84 | 0.76 | 0.83 | 0.28 | 0.40 | 0.25-0.54 | 0.69 | 0.32 |
| 26 ≤ | 0.89 | 0.97 | 0.94-0.99 | 0.37 | 0.24-0.50 | 0.84 | 0.77 | 0.83 | 0.29 | 0.42 | 0.27-0.56 | 0.70 | 0.34 |
| 27 ≤ | 0.88 | 0.96 | 0.94-0.99 | 0.39 | 0.26-0.52 | 0.85 | 0.75 | 0.83 | 0.31 | 0.42 | 0.28-0.57 | 0.68 | 0.35 |
| 28 ≤ | 0.88 | 0.96 | 0.93-0.99 | 0.39 | 0.26-0.52 | 0.85 | 0.72 | 0.83 | 0.31 | 0.41 | 0.27-0.56 | 0.64 | 0.35 |
| 29 ≤ | 0.87 | 0.95 | 0.92-0.98 | 0.41 | 0.28-0.54 | 0.85 | 0.71 | 0.83 | 0.32 | 0.42 | 0.28-0.57 | 0.63 | 0.36 |
| 30 ≤ | 0.86 | 0.95 | 0.91-0.98 | 0.46 | 0.33-0.60 | 0.86 | 0.71 | 0.84 | 0.37 | 0.47 | 0.33-0.61 | 0.63 | 0.41 |
| 31 ≤ | 0.85 | 0.94 | 0.90-0.97 | 0.46 | 0.33-0.60 | 0.86 | 0.68 | 0.83 | 0.37 | 0.45 | 0.31-0.59 | 0.58 | 0.40 |
| 32 ≤ | 0.85 | 0.94 | 0.90-0.97 | 0.46 | 0.33-0.60 | 0.86 | 0.68 | 0.83 | 0.37 | 0.45 | 0.31-0.59 | 0.58 | 0.40 |
| 33 ≤ | 0.83 | 0.92 | 0.88-0.96 | 0.50 | 0.37-0.63 | 0.87 | 0.64 | 0.83 | 0.40 | 0.46 | 0.32-0.60 | 0.54 | 0.42 |
| 34 ≤ | 0.82 | 0.92 | 0.88-0.96 | 0.52 | 0.39-0.65 | 0.87 | 0.65 | 0.83 | 0.41 | 0.47 | 0.34-0.61 | 0.55 | 0.44 |
| 35 ≤ | 0.82 | 0.91 | 0.88-0.95 | 0.52 | 0.39-0.65 | 0.87 | 0.64 | 0.83 | 0.41 | 0.46 | 0.33-0.60 | 0.53 | 0.43 |
| 36 ≤ | 0.80 | 0.90 | 0.86-0.95 | 0.56 | 0.42-0.69 | 0.88 | 0.63 | 0.83 | 0.45 | 0.48 | 0.34-0.61 | 0.52 | 0.46 |
| 37 ≤ | 0.79 | 0.89 | 0.84-0.93 | 0.57 | 0.44-0.71 | 0.88 | 0.60 | 0.82 | 0.46 | 0.47 | 0.34-0.60 | 0.48 | 0.46 |
| 38 ≤ | 0.77 | 0.87 | 0.82-0.92 | 0.57 | 0.44-0.71 | 0.88 | 0.56 | 0.81 | 0.45 | 0.44 | 0.31-0.58 | 0.44 | 0.45 |
| 39 ≤ | 0.76 | 0.86 | 0.81-0.91 | 0.57 | 0.44-0.71 | 0.88 | 0.53 | 0.79 | 0.44 | 0.42 | 0.29-0.55 | 0.40 | 0.43 |
| 40 ≤ | 0.75 | 0.86 | 0.81-0.91 | 0.61 | 0.48-0.74 | 0.88 | 0.55 | 0.80 | 0.48 | 0.45 | 0.32-0.58 | 0.42 | 0.47 |
| 41 ≤ | 0.75 | 0.86 | 0.81-0.91 | 0.63 | 0.50-0.76 | 0.89 | 0.56 | 0.81 | 0.50 | 0.46 | 0.34-0.59 | 0.43 | 0.49 |
| 42 ≤ | 0.74 | 0.85 | 0.79-0.90 | 0.65 | 0.52-0.78 | 0.89 | 0.55 | 0.80 | 0.52 | 0.46 | 0.33-0.59 | 0.42 | 0.49 |
| 43 ≤ | 0.73 | 0.85 | 0.79-0.90 | 0.69 | 0.56-0.81 | 0.90 | 0.56 | 0.81 | 0.57 | 0.49 | 0.37-0.62 | 0.43 | 0.53 |
| 44 ≤ | 0.72 | 0.85 | 0.79-0.90 | 0.70 | 0.58-0.83 | 0.91 | 0.57 | 0.81 | 0.59 | 0.51 | 0.38-0.63 | 0.44 | 0.55 |
| 45 ≤ | 0.72 | 0.84 | 0.79-0.89 | 0.70 | 0.58-0.83 | 0.91 | 0.56 | 0.81 | 0.59 | 0.50 | 0.37-0.62 | 0.43 | 0.54 |
| **46 ≤** | **0.70** | **0.83** | **0.78-0.88** | **0.74** | **0.62-0.86** | **0.92** | **0.56** | **0.81** | **0.63** | **0.51** | **0.39-0.63** | **0.43** | **0.57** |
| 47 ≤ | 0.68 | 0.80 | 0.75-0.86 | 0.74 | 0.62-0.86 | 0.92 | 0.52 | 0.79 | 0.62 | 0.47 | 0.35-0.59 | 0.38 | 0.54 |
| 48 ≤ | 0.65 | 0.77 | 0.71-0.83 | 0.74 | 0.62-0.86 | 0.91 | 0.48 | 0.76 | 0.60 | 0.42 | 0.30-0.54 | 0.33 | 0.51 |
| 49 ≤ | 0.64 | 0.76 | 0.69-0.82 | 0.76 | 0.65-0.87 | 0.92 | 0.47 | 0.76 | 0.62 | 0.42 | 0.30-0.54 | 0.32 | 0.51 |
| 50 ≤ | 0.62 | 0.73 | 0.67-0.80 | 0.76 | 0.65-0.87 | 0.91 | 0.45 | 0.74 | 0.61 | 0.40 | 0.28-0.51 | 0.29 | 0.49 |
| 51 ≤ | 0.60 | 0.70 | 0.64-0.77 | 0.78 | 0.67-0.89 | 0.92 | 0.43 | 0.72 | 0.63 | 0.37 | 0.26-0.49 | 0.26 | 0.48 |
| 52 ≤ | 0.59 | 0.70 | 0.63-0.76 | 0.78 | 0.67-0.89 | 0.92 | 0.42 | 0.71 | 0.62 | 0.37 | 0.25-0.48 | 0.26 | 0.47 |
| 53 ≤ | 0.57 | 0.68 | 0.61-0.74 | 0.78 | 0.67-0.89 | 0.91 | 0.41 | 0.70 | 0.61 | 0.34 | 0.23-0.45 | 0.24 | 0.45 |
| 54 ≤ | 0.54 | 0.63 | 0.56-0.70 | 0.78 | 0.67-0.89 | 0.91 | 0.38 | 0.67 | 0.59 | 0.30 | 0.19-0.41 | 0.20 | 0.41 |
| 55 ≤ | 0.52 | 0.61 | 0.54-0.68 | 0.78 | 0.67-0.89 | 0.91 | 0.37 | 0.65 | 0.58 | 0.28 | 0.17-0.38 | 0.18 | 0.39 |

*Note*. Level = Level of test (% of participants meeting the cut-off); 95% CI (Sen) = 95% confidence interval for sensitivity; 95% CI (Spe) = 95% confidence interval for specificity; PPV = Positive predictive value; NPV = Negative predictive value; ĸ(0) = Quality of specificity; ĸ(0.5) = Quality of efficiency; 95% CI (ĸ(0.5)) = 95% confidence interval for the quality of efficiency; ĸ(1) = Quality of sensitivity; Youden = Youden’s index.
